# Supplementary material for: Learning curve and surgical outcome of robotic assisted colorectal surgery with ERAS program
Source: Sci Rep. 2022 Nov 29;12:20566. doi: 10.1038/s41598-022-24665-w (PMC9709162; doi:10.1038/s41598-022-24665-w)
Supplement: Supplementary file 1 — Supplementary Information 1. [file 41598_2022_24665_MOESM1_ESM.zip › LSC Raw data-Ñ[▒K/096/H_ñw╝╨░O▒Kñσ.pdf]

正式摘要(電子簽章：000000200890239)

W52 / W52 ☐英文標題(注意:無英文姓名-請補病人基本檔!)

☐【讀取】憑證

臺中榮民總醫院
出院病歷摘要

轉入號 : 轉出號 :

[redacted]

(8)流水編號	
(9)入院日期	109/10/06 (2020) 直腸外科 W52 - 112
(10)轉科日期	
(11)出院日期	109/10/13 (2020) 住院天數計 7 日

診斷

- 入院 :Colon cancer at proximal T-colon
- 出院 :Malignant tumor of proximal T colon s/p laparoscopic right hemi-colectomy 2020/10/07

主訴

- Dyspnea for 6 months

病史

- This 79-year-old male has past history of
  1.Mildly coroanry ASHD
  2.Moderate AR, moderate MR, LVEF-56%
  3.Anemia, suspected iron deficiency
  4.Hypertension
  5.Brain stem hemorrhage s/p medical treatment.
  He complained chest tightness recently. He ever went to 光田 hospital where there was abnormal finding via perfusion scan. SOB was noted while walking. For second opinion, he went to CVS OPD for further management. Irregular heart beat and diastolic heart murmr (gr. 2-3) were noted. Microcytic anemia was suspected and blood transfusion was administered. As anemia maintained and stool test showed OB+, he was referred to GI OPD. CT revealed moderate wall-thickening and enhancement of proximal T-colon(axial:23-24), Colon Ca. may be compatible. Thus he was referred to our OPD. The patinet denied tarry nor bloody stool. There was no dizziness, poor appetite nor body weight change. Due to above reason, the patinet was admitted to our ward for laparoscopic right hemicolectomy with ERAS and further management.

體檢發現

- HEPATIC FLEXURE CA
  - General appearance :
    - easy looking , conscious clear , without cardiopulmonary distress
    - Vital signs自動帶入date 體溫(BT) 脈搏(P) 呼吸(R) 血壓(BP) 身高(BH) 體重(BW) BMI
      20201006 37.1℃ 102次/分 19次/分 156/104mmHg 159.8cm 62.1kg 24.3
    - Vital signs: B.P. 156/104 mmHg , P.R. 102 /min , R.R. 19 /min , B.T. 37.1 ℃
  - HEENT :
    - Eyes: no anemic conjunctiva, no icteric sclera, normal EOM
    - ENT: no otorrhea, no rhinorrhea, no erythematous mucosa, no injected tonsil, no deformity
  - Neck: supple, freely movable, trachea midline,no deformity, no engorged jugular vein , no palpable thyroid or lymph node enlargement
  - Chest:
    - Inspection: Grossly normal, symmetric and fully expansion, no accessory muscle used, no operation scar, no spider angiomata, no gynecomastia
    - Palpation: no palpable mass or nodule, no subcutaneous emphysema, no tenderness
    - Ausculation: clear breathing sound
  - Heart:

-
    - Palpation: no thrill, no heave
    - Percussion: normal heart size
    - Ausculation: regular heart beat, normal S1, S2, no gallop, no murmur
  - Extremities: No deformity, full ranged of motion without limitation, no clubbing finger or nail cyanosis, no pitting edema, no ulcer or pigmentation, no obvious skin lesion, no varicose vein, no bone, joint or muscle tenderness

CRS CONDITION

- Karnofsky performance status: (90) Able to carry on normal activity; minor signs or symptoms of disease
- PE of Abdomen:
- __1. Inspection: normal
- __2. Ausculation: normactive
- __3. Palapation: normal
- __4. Percussion: normal
- CEA: 7.04 ng/ml
- Imaging Studies:
- Colonoscope report: Transverse colon: huge tumor with easy touch bleeding near hepatic flexion
- Figures:
    1. colon & rectum

手術日期及方法

- 手術日期及方法20201007 Laparoscopic right colectomy and anastomosis

住院治療經過

- Operation was done on 10/07. Foley catheter was removed on 10/9 but acute urine retension was noted. Foley catheter was re inserted and harnalidge was added.Foley catheter was removed on 10/12. He can tolerance soft diet now. Under stable condition, we arranged discharge for him.

合併症

一般檢查

- 累積報告：

DATE	TIME	ALB	TP	BIL.T	BIL.D	ALKP	AST	ALT	LDH	CHOL	TG	HDL-C	LDL	UA	NH3	AMY
1091006	1732	4.0		0.24				20								
1091007	0927		6.8							108	58		52	6.1		
1091007	1744	3.4		0.42				19								

DATE	TIME	WBC	RBC	HGB	HCT	MCV	PLT	BLST	NEUT	BAND	SEG	LYM	MON	EOS	BAS
1091006	1724	5700		12.0	36.9		185		62.2			21.1	6.1	9.5	1.1
1091007	1742	9210		10.5	31.9		165		87.8			8.0	2.9	1.0	0.3

DATE	TIME	NA	K	CL	CA	BUN	CREAT	Mg	P	CRP	ESR	PCT	LIPASE	LACTATE
1091006	1732	137	4.4		8.8	26	1.77							
1091007	0927									0.088				
1091007	1744	142	3.3		7.8	20	1.29							

DATE	TIME	GLUCOSE	PAB	TRF	ZN	CU	KETONE	CK	CKMB	PT-P	PT-C	APTT-P	APTT-C	TRO-T
1091006	1716									10.3	10.3	29.1		

特殊檢查

病理報告

其它

出院時情況

- 3.改門診治療

出院指示

藥名	劑量	劑量單位	用法	頻次	數量	數量單位	現況	天數	單位
MOSAD tab 5mg	1	TAB	PO	TID	21	TAB	TH	7	天
OCAS Harnalidge 0.4 mg	1	TAB	PO	QDAC	7	TAB	TH	7	天
Ulstop tab 20mg	1	TAB	PO	QD	7	TAB	TH	7	天
Lactam tab 500mg	1	TAB	PO	QID	28	TAB	TH	7	天
TramTOR cap 50mg	1	CAP	PO	Q6HPRN	12	CAP	TH	3	天
Chlorzoxazone-250	1	TAB	PO	TIDPRN	9	TAB	TH	3	天

出院計劃

主治醫師 2062F 蔣鋒帆 / 住院醫師 9972C 陳智翔

正式摘要(**電子簽章：000000200890239)**

W52 / W52

doctorRank:B
